# Dentist education and labour market in Mexico: elements for policy definition

**DOI:** 10.1186/1478-4491-10-31

**Published:** 2012-09-13

**Authors:** Luz María González-Robledo, María Cecilia González-Robledo, Gustavo Nigenda

**Affiliations:** 1 Universidad Autónoma del Estado de Morelos, Calle Leñeros esquina Iztaccíhuatl s/n Col. Volcanes, CP 62350, Cuernavaca, Morelos, México

**Keywords:** Dentists, Education, Labor markets, Mexico

## Abstract

**Background:**

Here, the educational and labour market characteristics of Mexican dentists are revised. Dentistry is a health profession that has been scarcely studied in developing countries. This analysis attempts to understand the relationships and gaps between the supply and demand of dentists in the country. Around 5000 new dentists graduate every year looking for a place in the labour market.

**Methods:**

A cross-sectional study with exploratory, descriptive and correlational scope was carried out between 2006 and 2008. Analyses of quantitative data on dentists from national surveys and occupational statistics were complemented with qualitative information provided by 43 key informants in five Mexican states.

**Results:**

The 2008 dentist labour market can be characterized as follows: 75% worked in the private sector, most of them independently; more than two-thirds were women; the proportion of specialists was low (slightly more than 10%); unemployment was more than 20% and labour wastage was nearly 40%, with most wastage corresponding with female dentists. The increase in the number of dentists entering the labour market during the last two decades is more related to the educational market than to the population’s health needs and the number of dentists actually required to meet them.

**Conclusions:**

The problems identified in the Mexican dentist labour market necessitate urgent intervention on behalf of regulatory bodies in order to balance the tendencies of supply and demand in the number of trained professionals as well as in their incorporation into different market areas. Adequate policies are required to increase the likelihood of achieving this objective.

## Background

*The Toronto Call to Action 2006–2016: Towards a Decade of Human Resources for Health in the Americas*[1], establishes that “human resources are the basis of the health system” and that the contribution of this work force is a key factor in the ability of health systems to ensure equitable access to quality services for the entire population.

The Pan American Health Organization and World Health Organization (WHO) [2] state that the health systems of many different countries in the Latin American and Caribbean region presently face challenges derived from a series of problems related to human resources. Among other challenges, Pan American Health Organization and WHO highlight the lack of health personnel in some occupational categories; the lack of connection between the supply of health personnel and the labour market; the lack of personnel to address the health needs of large sectors of the population; the lack of workforce mobilization planning to prevent the well-known consequences of migration between public and private and rural and urban areas, and between different health system subsectors; poor working conditions; lack of motivation and low productivity of health personnel; inefficiency in resource allocation; and imbalance in the composition and distribution of the workforce.

Dentists are also affected by many of these problems that are common to most countries in the region. In most of these countries, oral health has been pushed into the background by diverse factors, including the scarcity of resources in competition with other areas defined by governments as high priorities (namely, infectious and/or chronic disease care), and by the lack of visibility of the pernicious consequences of oral diseases in the health of populations. Other aspects playing a role are the lack of clear policies and plans for oral health and insufficient commitment of educational institutions to train dentists in a way that responds to population oral health needs and demands, that is, with an emphasis on the prevention of high prevalence conditions such as cavities and periodontal disease.

In Mexico, dentists’ professional practice continues to be characterized by private independent practice, in spite of changing tendencies in the health labour market in most of the world [3]. By contrast, in Latin America in general, a progressive reduction of autonomous independent professional practice has been observed, alongside an increase in public institutional work and professional group practice [4]. There is also an increasing tendency towards subcontracting, which has led to stagnation of hiring for formal public sector jobs, increased flexibility of working conditions and more precarious and unstable employment [5-8]. A major factor reinforcing independent private dental practice in Mexico is the type of training they typically receive. Dentistry schools and universities have long been characterized by curricula with a strong biomedical component focusing on restorative and curative aspects, which encourages individual work in opposition to the public health approach that has been recommended by health international agencies to successfully tackle the consequences of demographic and epidemiological changes.

Health workers in the United Mexican States face serious imbalances related to the interaction between the educational and labour markets. For many years, the main stakeholder has been the government and its agencies but in the last 20 years a broad variety of institutions have been playing an increasing role in education and employment. Dentistry has a special status. It differs from other health professions because, despite government’s involvement in strategic decisions, the labour market has remained mainly private. At the same time, private schools have grown in influence and dentistry as a whole has very little participation in the development of public health activities.

By presenting a panorama of the dentist education and labour markets in Mexico, we aim to generate evidence to promote an effective dialogue among all involved institutions, thereby providing a foundation for policy development of this important health resource and positively impacting the oral health of the Mexican population.

## Methods

The results presented in this document are part of a broader research project called “Training, Employment and Regulation of Human Resources in Health: Bases for Strategic Planning”, carried out at the National Institute of Public Health of Mexico with funds from the National Council of Science and Technology. This project focuses on five key health occupations: physicians, dentists, licensed nurses, auxiliary nurses and pharmacists.

This is a cross-section study which relies on the use of mix methods (quantitative and qualitative) of research. The main objective of mix methods is to integrate and obtain a broader perspective of the problem under study to better understand the phenomenon [9,10]. “Using multiple approaches can capitalize on the strengths of each approach and offset their different weaknesses. It could also provide more comprehensive answers to research questions, going beyond the limitations of a single approach” [11]. The use of mix methods allowed us to broaden the understanding of training patterns, the labour market and regulatory issues of dentists in Mexico. The presented results emerged initially from the analysis of quantitative data; however, the analytical capacity to understand the role of context variables was facilitated by the use of qualitative data. The fieldwork was carried out between 2006 and 2008.

The quantitative component examining student enrolment, graduation and degrees in dentistry careers used secondary sources including the *2007 Catalogue of Bachelor’s Degree Careers at Universities and Technological Institutes* of the National Association of Universities and Institutions of Higher Education (ANUIES), and the statistical yearbooks published by the same institution from 1990 to 2004. Quantitative information on dentist employment was obtained from two databases: the 2000 National Employment Survey (ENE in Spanish) and its successor, the 2008 National Occupation and Employment Survey (ENOE in Spanish). Both surveys were carried out by the National Institute of Statistics and Geography (INEGI in Spanish). According to the information in these surveys, there were 93 557 dentists in Mexico in 2000 and 117 449 in 2008 (Table 1). The survey excluded people younger than 22 years and older than 65 years; the former group was excluded because 22 years old is considered to be the minimum age at which a university degree can be obtained, while the latter group was excluded to avoid over estimating unemployment.

**Table 1:** Characteristics of dentist labour market in Mexico, 2000–2008

**Characteristic**	** 2000**	**2008**
**Total (absolute number)**	93 557	117 449
	** %**	**%**
**Sex**		
Women	60.5	67.3
Men	39.5	32.7
**Training level**		
Bachelor’s degree	95.2	88.9
Postgraduate degree	4.8	11.1
**Work status**		
Employed	81	78.9
Unemployed	19	21.1
**Employed**		
In his/her area of training	72.8	78.9
In a different area of training	27.2	21.1
**Geographic location in his/her area of training**		
Urban	97.7	96.5
Rural^a^	2.3	3.5
**Employment sector in his/her area of training**		
Private	88.2	75
Public	11.8	25
**Number of jobs held**		
One	93.9	89.7
Two	5.8	10.3
Three	0.3	0

*Source: 2000–2008 National Occupation and Employment Survey database*.

^a^Rural: Localities with less than 2500 inhabitants.

The analysed variables included characteristics of training (age, sex, enrolment, terminal efficiency) and the labour market (employment status, position at work, employment sector, geographical distribution). Other elements of analysis included subordinate and paid work, the context in which individuals enter a job as well as the circumstances under which they leave it, job searching, and the measurement of unemployment rates in accordance with standards established by the Organization for Economic Co-operation and Development. A subsequent analysis estimated the variable known as labour wastage. This term refers to qualified personnel who do not perform activities related to their formal education, due either to unemployment or because they work in areas other than that in which they received their formal training [12]. Labour wastage has been estimated in previous studies for physicians [12] and nurses [13].

The quantitative analysis was exploratory, descriptive and correlational. Inferences were made based on the entire population of dentists throughout the country [10] using the Stata statistical package, version 9. The employment surveys used for the study (carried out in 2000 and 2008) are not comparable for all variables. However, the variables chosen for comparison among the groups of dentists were carefully selected to ensure their comparability.

A qualitative component was carried out in five states: Baja California at the northern border, Michoacán in the Pacific region, Campeche in the southeast and San Luis Potosi in the central-northern region. The central region was represented by the Federal District (Mexico City), which was also the site of the pilot test. The main inclusion criteria were that these states have professionally recognized higher learning institutions representing different geographic regions in the country and at least one actively hiring public health institution.

Data were collected for this component via literature searches and semi-structured interviews with key informants. A key informant was defined as an individual that, given his or her labour background, experience and institutional position, could offer informed opinions about the topics under investigation. In the selection of these informants we attempted to identify individuals occupying top institutional positions using the “snowball technique” according to criteria established by Doreian and Woodard [14]. Informants were selected from public and private training, employing and regulatory institutions, and from dentistry associations. Of 43 total key informants, 18 occupied top positions at educational institutions, 9 occupied positions in employment institutions and 16 belonged to regulatory institutions. The sample was distributed geographically as follows: Baja California contributed 8 informants, Campeche 9, Distrito Federal 5, Michoacán 7 and San Luis Potosí contributed 14 informants.

Seven interview guides were designed with questions that varied according to the type of key informant. Informants belonging to higher education institutions were questioned about available resources for education, educational curricula and programmes, quality of educational processes, profiles of graduates, and options available for graduates for finding a paid job (see Table 2).In the case of informants classified as employers, interviews emphasized aspects related to the incorporation of dentists into the labour market, recent recruitment tendencies, the type of hiring taking place, and working conditions. Regulatory institution informants were questioned about their capacity to apply regulations, the mechanisms used for this purpose, and the impact of regulation on training and labour markets.

**Table 2:** Types of key informants and interview topics explored (qualitative component)

**Type of key informant**	**Category**	**Specific topic**
Federal Secretary of Health decision-makers	Training	· Human health resources training policy
		· National HR (dentists) planning
		· Coordination with other institutions
		· HR (dentists) geographical distribution
	Labour market	· Dentists’ national need assessment
		· Dentists’ contracting policies in Mexico
State Ministry of Health decision-makers	Training	· Role of educational institutions in dentist training
	Labour market	· Role of institutional employers in dentist labour market (contracting, working conditions, salaries etc.)
	Regulation	· Role of collective actors (councils, colleges, associations, etc.) in the regulation of dentists
Social Security Institutions (IMSS and ISSSTE) decision-makers	Training	· Role of IMSS and ISSSTE in HRHtraining
		· Match between graduate profile and labour market
		· Role of IMSS and ISSSTE as HRH employers
	Labour market	· Health personnel hiring methods
		· Health personnel development
		· Education policies
Decision-Makersat Public and at Public and Private Educational Institutions	Training	· Curricula
		· Terminal efficiency
		· Graduates
		· Graduate profile
	Labour Market	· Match between graduate profile and labour market
		· Graduate entry into labour market
		· Working conditions
		· Professional certification (characteristics, periodicity)
	Regulation	· Program accreditation
		· Interference in working arrangements

HRH: Human resources for health; IMSS: Mexican Institute of Social Security; ISSSTE: Institute of Security and Social Services for State Workers.

To test the precision and validity of the instruments, pilot tests were conducted with employer informants from the Secretary of Health, the Mexican Institute of Social Security and the Institute of Security and Social Services for State Workers. All informants granted informed consent. All interviews were taped on audio, transcribed and processed for systematization with the Atlas Ti software package.

All analytical categories were defined prior to data processing. Specifically, the coding classifications used for conceptual ordering of information collected during the interviews were training characteristics and determinants; working conditions; hiring practices; unemployment; under employment; and illegal practice [14].

## Results

### Training

According to ANUIES, in 2007 there were 75 schools of dentistry in Mexico, of which 37 were public and 38 private [15]. Between 1990 and 2004, total enrolment in undergraduate dentistry programs increased by 28.8%, from 25 445 to 35 771 students. In 1990, 93% of enrolments corresponded to public schools and 7% to private, a tendency which had changed by 2003, when the public proportion of dentistry students nationwide decreased to 82%, with the remaining 18% enrolled in private schools.In terms of absolute enrolment, a sustained increase in private students is observed, from 1777 students in 1990 to 6193 in 2004, an increase of 71.3% enrolment at private schools. By contrast, enrolment at public schools increased by only 15.6% in the same period (23 668 and 28 046 students, respectively). An analysis of enrolment by sex shows that women have greater disposition to engage in dentistry training than men: on average, 6 of every 10 students enrolled were women [16].

**Figure 1:**
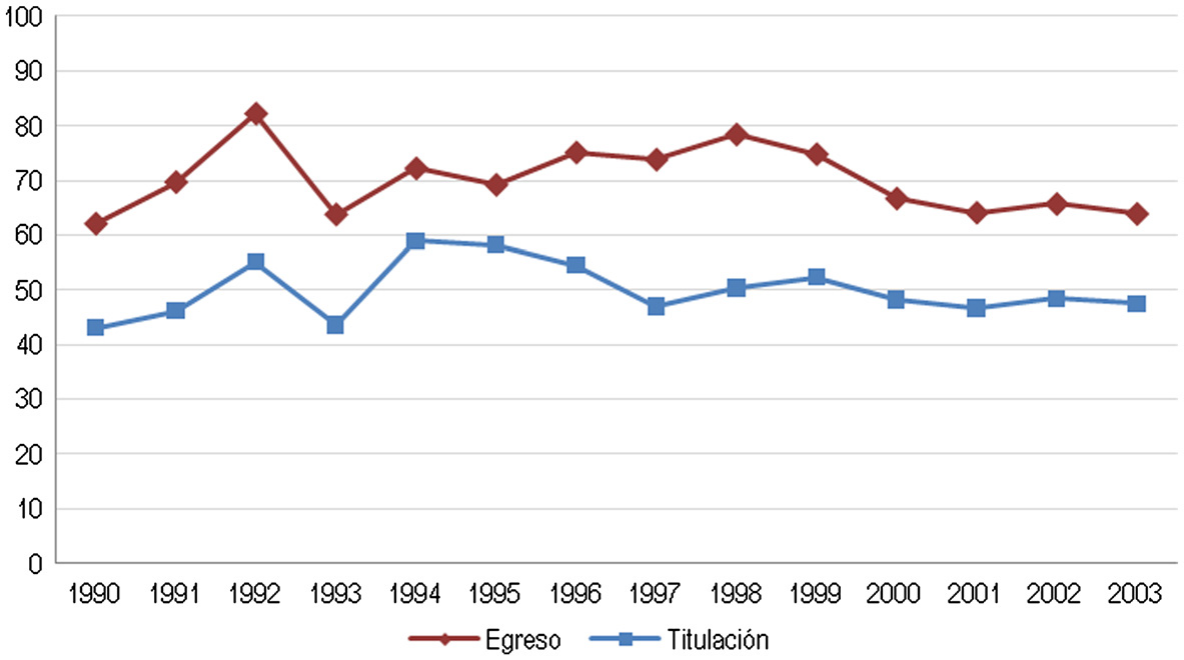
**Dentists’ terminal efficiency, 1990–2004.** Authors’ own estimations based on the National Association of Universities and Institutions of Higher Education statistical yearbooks (1990–2004).

Regarding terminal efficiency (the proportion of students in a five-year cohort who graduate), our findings show that the number of graduates (students who have completed all courses and credits) was much higher than the number of graduated students who went on to receive their degree and were subsequently granted a practicing license after completing the necessary procedures before the Federal Secretary of Education. An overall stable trend was observed over a 15-year period, although the difference between the two indicators tended to diminish in recent years. On average, about 70% of enrolled students graduated and only 50% of the cohort received their degree and practicing license (Figure 1). Key informants across the five states identified the following factors related to attrition in schools of dentistry: the scarcity of positions at public institutions, the lack of financial resources to pay fees and/or purchase materials and instruments for school, the lack of health units to be able to comply with the social service period (a requisite step to obtain a degree and subsequently, a practicing license) and the lack of financial resources to set up a consultation office once graduated. All of these factors, separately and in combination—in addition to the lack of real labour market regulation—lead to unlicensed individual practice. The following testimony states the factors underlying attrition in a local university:

“Around ninety students per class start the training but between thirty eight and forty obtain the degree. Various factors explain this level of attrition, among them the lack of commitment with training, the cost of fees and the price of equipment for practicing. They [the students] have to buy their own instruments although other materials are provided by the university.” Educator Campeche P.8

To legally practice dentistry in Mexico, it is mandatory to have a degree issued by an officially recognized university, to have a professional license bestowed by the Federal Secretary of Education’s General Directorship of Professions, and to be registered with the health authorities [17-19]. Despite this regulation it is common to observe a considerable number of dentists in private practice who have graduated from universities but have not fulfilled the degree and licensing requirements [20-22]. This issue can be explained by the lack of mechanisms to enforce professional legal practice, as stated by acouple of our key informants.

“While still at school, students look for working options (.) Graduate dentists owning a clinic employ students and recent graduates. These students attend the clinics in the afternoons to perform different activities but in the mornings they can use the clinicÂ´s equipment to provide services to their own patients.” Educator Campeche P.8

“Many of them (undergraduate) adapt their garages at home to set up a small practices and save Money.” Regulator. San Luis Potosí P.26

Between 2000 and 2008, a 20.3% increase was observed in the total number of dentists in the labour market. This reflects an increase in the number of graduates and in the number of schools, especially in the private sector. The 1980 Constitutional Reform raised university autonomy to constitutional level in Mexico, meaning that higher education institutions could be created as long as they comply with all legal requirements without consideration for the actual number of students to be trained in each profession. Indeed, most schools fail to define *numerus clausus*. Informants throughout the states also pointed out that the increase in the number of dentists is not based on the population’s needs, the number of dentists the country requires, nor an analysis of the prevailing labour market. Instead, the increase is due to factors derived from a social demand for higher education and the capacity to pay off those students that attend the rising number of private schools. Higher education institutions can maintain a certain number of students based on factors such as facility-based institutional capacity (number of classrooms and dental units), the number of practice positions available for students, and the availability of teachers. Thus, according to informants, along with the social demand for education, schools respond more to the resources available to carry out the training than to population-based oral health-care needs or institutional demand for dentists, as is confirmed by the following testimonies:

“More than thinking on how many dentists does the state need, how many of them are practicing or how many are trying to open a clinical practice (.), we need to think how many students we are going to train every year.” Educator. Campeche P.8

### Labour market

**Figure 2:**
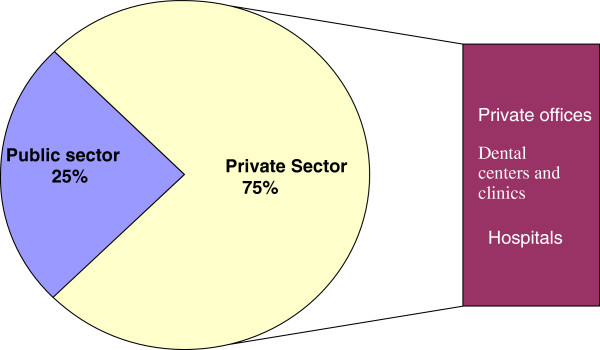
**Institutions employing dentists in Mexico, 2008.** Source: 2008 National Occupation and Employment Survey (ENOE), National Institute of Statistics and Geography (INEGI).

Table 1 demonstrates key dynamics in market composition between 2000 and 2008. The proportion of women in the market increased from 60.5% to 67.3%, while the percentage of dentists who are specialists more than doubled, from 4.8% in 2000 to 11.1% in 2008. As with other occupational groups in the health sector, dentists are presented with a variety of options when entering the labour market, including public sector, social security, private sector, and academic. However, as opposed to doctors and nurses, who mainly can find a position in a public or social security institution, the main option for most dentists is the private sector, as shown in Figure 2.

**Figure 3:**
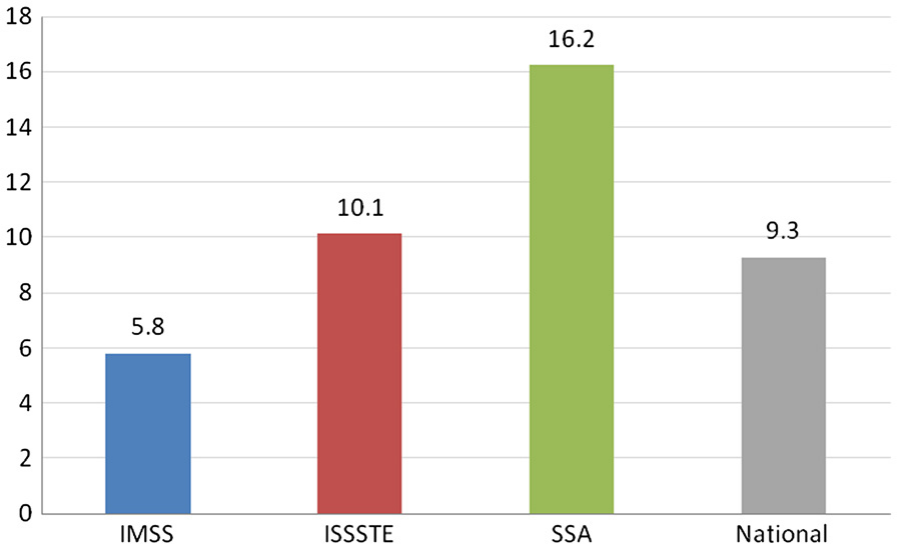
**Distribution of dentists by institution, Mexico 2008.** Authors’ own estimations based on the 2008 National Occupation and Employment Survey (ENOE).

Of the total number of dentists employed in 2000, 88.2% were practicing in a private unit. By 2008, this percentage had dropped to 74.9% (Table 1 and Figure 3). Although the dentists’ labour market has traditionally been dominated by private individual practitioners, more recently salaried positions appear to be gaining importance. This new trend may increase in the future as public and private health-care institutions identify oral care as a relevant in the supply of their health interventions [23].

Interviewed employers perceive an excessive number of dentists in the country and assert that academic institutions train more resources than the country and health institutions require. This exacerbates the problems with job searching and placement of graduates in the labour market. However, while one side of the problem is the surplus of dentists, on the other side is the low demand for this professional group by public and social security institutions because oral health is not yet considered a priority (in 2008, only a quarter of dentists worked in public institutions). The following testimony vindicates this assessment:

“In hospitals there is no demand for dentists, even considering that, no doubt, the first cause of morbidity in the country are [sic.] oral diseases.” Employer Federal Ministry of Health P.16

**Figure 4:**
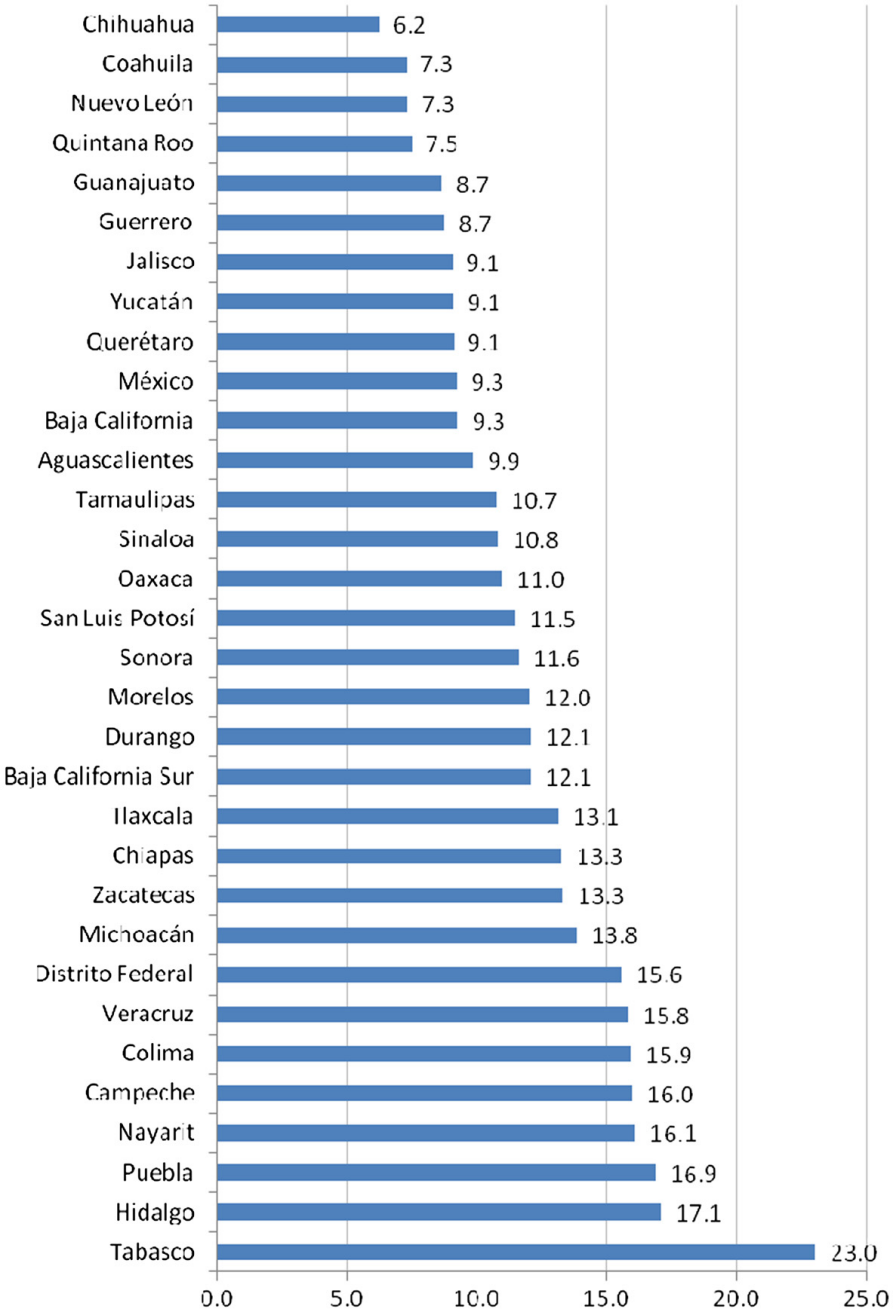
**Number of dentists per 100 000 inhabitants by state, Mexico 2008.** Authors’ own estimations based on the 2008 National Occupation and Employment Survey (ENOE).

Additionally, the evidence shows a distorted geographical distribution of dentists, with urban areas concentrating more than 95% of dentists across the country, creating disparities in the geographic availability of this profession (Table 1). However, the number of dentists relative to the population varies greatly by state, from 6.2 dentists per 10 000 inhabitants in the state of Chihuahua to 23 per 10 000 inhabitants in Tabasco (Figure 4).

With respect to employment status in 2008, 78.9% of dentists had employment (employed and underemployed included) while 21.1% were unemployed. Considerable unemployment among dentists is observed, with the proportion of unemployed dentists increasing during the period, albeit by only two percentage points.The opposite phenomenon is multi-employment. Although among dentists this is not very common, by 2008, 10.3% with employment declared having two jobs.

As for underemployment (meaning a dentist is employed but performs activities in a realm in which they did not receive formal training), an important decrease is observed between the two years under study: underemployment was 27.2% in 2000 and 21.1% in 2008. Quantifying underemployment is quite relevant since it is not only an indicator of the inability of the market to absorb supply, but also of the ability of graduates to place themselves in other markets that are indirectly related or completely unrelated to the profession they originally chose.

**Figure 5:**
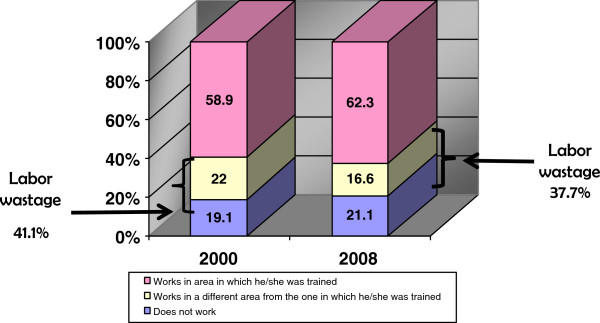
**Labour wastage for dentists in Mexico, 2000–2008.** Source: 2000 National Employment Survey (ENE) and 2008 National Occupation and Employment Survey (ENOE), National Institute of Statistics and Geography (INEGI).

Labour wastage for the year 2000 topped out at 41%, which decreased to 37.7% in 2008. These figures demonstrate a serious gender equity problem since most labour wastage occurred among women. There was also a slight increase in the number of women who did not work in 2008 (Figure 5).

Various informants related the causes of this situation;among others:

•Public, social security and private institution demand for dentists is quite low, and most of the available positions do not offer satisfactory pay nor attractive working conditions.

“We have students that after graduation are hired by public institutions as health promoters but not as dentists. Those who can obtain a position as dentists are very few.” Educator. Campeche P.8

•Existing incentives are insufficient to bring dentists to remote and/or rural areas.

•Data show an impressive concentration of dentists in metropolitan and urban zones, which increases competition among professionals; part of this competition comes from non- licensed dentists.

•A considerable proportion of female dentists decide to perform household duties. According to ENE data from 2000, 84% of women who did not work said it was because they were homemakers. According to 2008 ENOE data, this figure had decreased to 73%.

•According to testimonies of dental school directors, dentists are mainly trained to “set up their own business or office” and are encouraged to continue their training in some clinical specialty as stated by one of our key informants:

“At denstistry schools the students are trained to graduate and set up their own business because we know that in public institutions very few dentists are contracted.” Educator. Campeche P.8

## Discussion

This is one of the first attempts to analyse the educational, labour market, and regulatory patterns of dentists in Mexico using a mix-methods approach. Although our scope was intended to be broad, the diversity of the phenomena, its determinants and its consequences are too complex to be properly explored in a single study. Therefore, our explanatory capacity remains somehow general and only in very specific issues was it possible to have a deeper understanding. Many unexplored issues remain and they should be the subject of future research. Also the data that was used is clearly incomplete, leaving gaps of explanation in various areas. Educational and labour market statistical data represented the entire country (but not specific states) and were available for comparison across a certain period of time, while it was only possible to obtain qualitative data in five selected states. These methodological limitations should be initially recognized to develop a balanced interpretation of the results presented in the previous section.

It is generally accepted that labour markets reflect the ability of educational and labour institutions to merge their capacities to train and employ human resources for health both in the number of people trained and the type of skills obtained [24]. The dentist labour market in Mexico is clearly unbalanced as shown by the followingfeatures. Some of these features are present in other occupational health groups:

•Unemployment and underemployment are common, since positions in the public sector are scarce and a considerable proportion of dentists do not own the capital to open their own office.

•Labour wastage is quite high: more than 37% of the country’s dentists are unemployed or carrying out activities for which they were not trained. This is of concern because dentistry training represents a substantial economic and social investment —for the government as well as for households— that is not providing returns.

•Despite the lack of published evidence reporting the magnitude and distribution of dentists who work without having fulfilled the legally established requirements, illegal dentistry practice is a real phenomenon. Health authorities and representatives of regulatory institutions and associations and schools of dentistry acknowledge the existence of a broad group of non-license holding practitioners in the private sector.

These findings are not exclusive to dentistry practice in Mexico. There is published evidence of unemployment, underemployment, labour wastage, non-standardized working conditions, employment instability, precarious working conditions and salaries, and irregular professional practice in many other Latin American countries such as the Argentine Republic, the Bolivarian Republic of Venezuela, the Eastern Republic of Uruguay, the Federative Republic of Brazil, the Republic of Chile, the Republic of Ecuador, and the Republic of Peru [25-32]. Particularly in the Republic of Colombia [33], illegal practice has been identified as a major market problem and regulatory initiatives have been already put in place.

Also worth mentioning is the evidence of an increase in the placement of dentists in the public and social security sectors, alongside a decrease in private practice. This phenomenon may be associated with a recent increasing demand for public sector personnel by the Secretary of Health, which has incorporated some preventive and curative oral health interventions into the Popular Health Insurance’s package of benefits, which aims to provide funds to state-level public health-care systems to guarantee the provision of services to the population not covered by social security. In spite of this, informants from the Secretary of Health recognize that public health-care units lack adequately equipped dental offices that provide timely and high quality care [34,35]. Therefore, not all hired dentists are asked to carry out the normal activities of a dentist but different ones to which they need to adapt. This should be considered an expression of underemployment.

Educational and labour institutions dentistry policies follow differential paths. The first are dominated by private-sector objectives and preferences. While educational institutions are interested in training a number of students adequate to their available resources and infrastructure, public health institutions still not define oral health as a priority area, making them still a restricted market for dentists. In this scenario, dentists preferably find their location in private niches mainly working as solo practitioners having a direct interaction (clinical and economic) with clients. Currently, schools do not have an incentive to make a shift of technical capacities in the training of their students.

In addition to training-related difficulties, it is clear that there is a lack of leadership from a collective actor capable of providing systemic objectives for the definition of policies for training, the labour market, and regulatory issues. Furthermore, the particular characteristics of the labour market for dentists in Mexico underscore the urgency of defining policy and regulations in these areas. Policy and regulation should be defined in light of population needs, the requirements of health institutions as employers, and of the labour market, with the active participation of training institutions, governing authorities, service providers, dental associations, and other interested stakeholders.

The lack of proper policies and regulatory instruments of human resources in health leads to a set of negative consequences, such as lack of personnel; unmet demand; imbalances in geographical, labour and institutional distribution; excessive or insufficient qualifications of personnel; excessive or insufficient use of resources; desertions, unemployment or underemployment; and a delayed response in adapting to health-care trends (in terms of technology, new procedures, etc.). Thus, developing a human resource policy process in dentistry would allow the production of an adequate number of professionals with the knowledge, ability, attitude and ideal qualifications to take appropriate actions in the right place and at the right time to achieve established oral health objectives.

Some of the advantages of implementing policy in dentistry are:

•contributing to the improvement of the population’s oral health and of service provision, particularly in the public health sector;

•improving geographic distribution through strategies and programs that motivate dentists to stay in areas of high unmet need through the implementation of economic incentives, training scholarships, subsidies, etc.;

•gradually balancing the relationship between the education and labour markets; and the amount and quality of training, payment and work conditions (labour regulations) and performance.

Finally, to overcome some of the identified challenges and advance toward pre-established objectives, it is necessary to determine the volume, distribution and occupational profile of this resource according to the oral health needs and demands of the population; as well as improve the mechanisms and instruments that permit the regulation of the supply of this resource, including follow-up, monitoring, and evaluation of professional practice. An adequate planning process would help greatly in achieving this objective.

## Conclusions

The case analysed provides lessons for Mexico at both the federal and state levels, as well as for other countries, in at least the following three areas: the generation of evidence based on the use of national surveys, censuses and other sources of information available in the country; the identification of problematic areas requiring urgent intervention; and the use of information by the public and private stakeholders to formulate policies aiming at developing a better balance between supply and demand of dentists.

One relevant phenomena raised in the paper is illegal practice. This is a complex phenomenon that requires more research to be understood in depth. Illegal practice clearly shows that links between educational and labour market institutions go beyond the formal interaction. This type of phenomenon can not be fully understood only by the analysis of formal statistics. New ways of research should be developed to achieve this understanding.

Policy and regulation are key functions to obtain a fair balance between the production and demand of dentists. For the Mexican case, unemployment, underemployment and illegal practice are three undesired consequences of the lack of policy and regulation. Also, despite recent efforts, the fact that oral health has not been considered a priority in public institutions [36] has led the demand to be driven by private forces creating a labour market characterized by independent, fee-for-service, restorative practice. This type of practice does not support WHO recommendations [37] to base oral health on promotion and prevention practices.

Finally, in Mexico, regulation of health professions in the last 20 years has been transferred from state authorities to professional groups that pursue the development of a fully professional practice and the improvement of quality of performance. Doctors and nurses have responded positively, defining and making responsible those groups to carry out regulation on behalf the government. In the case of dentists, professional associations have not been able to successfully claim this attribute, leaving the market to be dominated by the interests of stakeholders that do not represent the profession.

## Competing interests

The authors declare that there are no competing interests.

## Authors' contributions

LMG and MCG processed and analysed information and participated in writing and editing the article. GN designed the overall study and the data collection instruments and contributed to information analysis and drafting the article, as well as its technical review. All authors have read and approved the final version of the manuscript.

## Authors’ information

LMG is a Researcher/Professor at the Morelos State Autonomous University (Mexico); MCG is a researcher at the National Institute of Public Health (Mexico); GN is researcher at the National Institute of Public Health (Mexico).
